# Prognostic Value of Serum Insulin‐Like Growth Factor‐1 in Patients With Anal Fistula Treated by Incision‐Thread‐Drawing Surgery

**DOI:** 10.1002/ags3.70037

**Published:** 2025-05-07

**Authors:** Zhijun Wu, Xuexue Yin, Jing Li

**Affiliations:** ^1^ Department of Traditional Chinese Medicine Zibo Central Hospital Zibo Shandong China; ^2^ Department of Gynecology Zibo Central Hospital Zibo Shandong China

**Keywords:** anal fistula, incision‐thread‐drawing surgery, insulin‐like growth factor‐1 (IGF‐1), wound healing

## Abstract

**Background:**

Anal fistula is a chronic condition characterized by an abnormal tract between the anal canal and perianal skin, often leading to infection, inflammation, and impaired quality of life. Incision‐thread‐drawing surgery is the main treatment for anal fistula. However, the risk of poor postoperative healing remains significant. This study investigates the prognostic value of preoperative serum insulin‐like growth factor‐1 (IGF‐1) levels in predicting wound healing after incision‐thread‐drawing surgery for anal fistula.

**Methods:**

A total of 129 patients undergoing incision‐thread‐drawing surgery for anal fistula were enrolled. Patients were divided into a healing group (*n* = 87) and a non‐healing group (*n* = 42) based on wound healing status at 1 month post‐surgery. Serum IGF‐1 levels were measured preoperatively, and their association with wound healing, inflammatory cytokines, and postoperative anal function was analyzed using logistic regression, receiver operating characteristic (ROC) analysis, and Wexner Incontinence Score.

**Results:**

Preoperative serum IGF‐1 levels were significantly lower in the non‐healing group (*p* < 0.001). IGF‐1 levels above 174.9 ng/mL were associated with better wound healing (OR = 0.603, *p* = 0.005) and lower postoperative inflammation. Higher IGF‐1 levels correlated with improved anal function at 7 and 14 days post‐surgery (*p* < 0.01).

**Conclusion:**

Preoperative serum IGF‐1 levels are a valuable prognostic biomarker for predicting wound healing and postoperative recovery in patients undergoing incision‐thread‐drawing surgery for anal fistula, potentially guiding clinical decision‐making and patient management strategies.

## Graphical abstract

IGF‐1 levels above 174.9 ng/mL were associated with better wound healing and lower postoperative inflammation. Higher IGF‐1 levels correlated with improved anal function at 7 and 14 days post‐surgery.

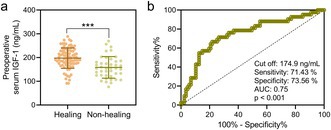

## 1 Introduction

An anal fistula is an abnormal epithelialized tract that forms between the anal canal and the perianal skin, often resulting from an infection in the anal glands that progresses to form an abscess and subsequently a fistulous tract [1, 2]. It is characterized by chronic inflammation, pain, purulent discharge, and potential fecal incontinence, significantly impacting a patient's quality of life [3, 4, 5]. The etiology of anal fistulas is multifactorial, involving factors such as cryptoglandular infections, Crohn's disease, trauma, radiation, malignancy, and tuberculosis [6]. Management options vary from conservative approaches to surgical interventions, such as fistulotomy, fistulectomy, seton placement, and advanced techniques like video‐assisted anal fistula treatment (VAAFT) [7, 8].

Currently, incision‐thread‐drawing surgery is a widely adopted clinical treatment for anal fistulas. However, the unique physiological and anatomical structures of the anal canal and surrounding tissues pose significant challenges for postoperative tissue repair. Surgical procedures often result in substantial tissue damage, including necrotic tissue formation, which complicates the healing process. The mechanical stress of postoperative defecation can further hinder tissue repair by expanding the wound and disrupting the regeneration of the affected area. Delayed or incomplete healing is a frequent outcome, contributing to chronic inflammation, prolonged recovery periods, and an increased risk of recurrence. Addressing the limitations of tissue repair in the context of anal fistulas is therefore critical for improving surgical outcomes [9].

Insulin‐like growth factor‐1 (IGF‐1), primarily produced by the liver and locally synthesized by various cell types such as fibroblasts, osteoblasts, and keratinocytes, plays a vital role in tissue repair and regeneration [10, 11]. Previous studies have explored the association between IGF‐1 and wound healing processes, demonstrating its role in promoting cell proliferation, migration, and extracellular matrix synthesis [12, 13]. However, research on the specific relationship between IGF‐1 and anal fistula healing is limited. Preclinical models suggest that IGF‐1 contributes to tissue repair in regions prone to chronic inflammation by enhancing fibroblast activity and collagen deposition, processes critical for fistula closure and tissue remodeling [14, 15]. Clinical studies have further indicated that elevated IGF‐1 levels correlate with improved healing outcomes in other types of surgical wounds, suggesting potential relevance for anal fistulas [16].

Notably, IGF‐1 has been implicated in enhancing the regenerative capacity of epithelial and connective tissues, which are key components involved in the healing of anal fistulas [17]. Its ability to activate intracellular signaling pathways through IGF‐1 receptors (IGF‐1R) has been shown to promote re‐epithelialization and reduce scar formation in wound models [18]. Additionally, IGF‐1's anti‐inflammatory properties may mitigate the chronic inflammation often observed in anal fistula cases, further supporting its therapeutic potential [19]. While direct evidence linking IGF‐1 to anal fistula repair remains sparse, these findings highlight its promising role in addressing the challenges of tissue repair and regeneration in this context.

Given its critical functions in tissue repair, this study aims to investigate the prognostic value of preoperative serum IGF‐1 levels in patients undergoing incision‐thread‐drawing surgery for anal fistulas.

## 2 Methods

### 2.1 Study Design and Participants

This study included 129 eligible patients who underwent incision‐thread‐drawing surgery for anal fistula treatment. Based on the healing status 1 month postoperatively, the patients were divided into two groups: the healing group (87 cases, with Grade A incision healing characterized by good wound healing without adverse reactions) and the non‐healing group (42 cases, with Grade B incision healing characterized by inflammation such as redness, swelling, and fluid accumulation at the wound site). The study was approved by Zibo Central Hospital. Written informed consent was obtained from the patients.

Inclusion Criteria: Patients met the diagnostic criteria for anal fistula as per the “Chinese Expert Consensus on Diagnosis and Treatment of Anal Fistula (2020 Edition)”; age ≥ 18 years; complete fistula formation with a length ≥ 3 cm; no history of previous anal fistula surgery; patients provided informed consent and agreed to follow‐up.

Exclusion Criteria: (1) Patients with special types of anal fistula caused by Crohn's disease; (2) Patients with other anal diseases such as hemorrhoids; (3) Patients with abnormal coagulation function; (4) Patients with severe liver, kidney, cardiovascular, cerebrovascular diseases, malignant tumors, or immune system diseases.

### 2.2 Surgical Procedure and Grouping

Clean enema was performed the night before the surgery. Lateral lying position was taken during the surgery and intraspinal anesthesia was conducted. After the anesthetic is satisfied, a 1.5 cm long radial incision was made at the external or the highest point of the abscess. Pus cavity was expanded by the middle curved forceps to fully drain the pus. After the abscess is fully drained, the probe was entered into the anus through the outer opening of the fistula, and protruded through the inner opening. The skin, subcutaneous tissue, and fistula between the probes were cut with an electric knife. Then infected anal sinus and anal gland were removed. The rubber band is guided by the probe from the high inner opening to the external opening to thread the deep fistula. The purulent secretions in the sinus were fully drained, and the necrosis in the sinus was removed. If the sinus is large and not deep, the thread was kept to assist drainage. If the sinus is deep and not suitable for incision treatment, the rubber band should be ligation and fixed after adjusting the tightness. The surgical margins were trimmed. Hemostasis, disinfection, and perianal compression bandaging were finally performed. To avoid subjective bias, the assessment of wound healing was performed by experienced clinicians following the wound healing evaluation criteria established by our department. Furthermore, the clinicians responsible for the wound healing evaluation were blinded to the overall study design and objectives.

### 2.3 Anal Function Scoring

Anal function was evaluated using the Wexner Incontinence Score at 1 and 2 weeks postoperatively, covering five aspects: gas, liquid stool, solid stool, use of pads, and lifestyle changes. The scoring ranged from 0 (never) to 4 (always), with a total score of 20 points. Higher scores indicate worse anal function.

### 2.4 Measurement of Serum Biomarkers

Serum levels of IGF‐1 (EH0165), IL‐8 (EH0205), IL‐1β (EH0185), and TNF‐α (EH0302) were measured using ELISA kits, purchased from Wuhan Fine Biological Technology Co. Ltd. The concentrations of vascular endothelial growth factor (VEGF) (EH0327) and TGF‐β (EH0287) in wound granulation tissue were also measured using ELISA kits from the same company.

### 2.5 Statistical Analysis

The data from this study were analyzed using SPSS 25.0. For normally distributed continuous data, means ± standard deviations ($\bar{x}\pm s$) were used, and comparisons between groups were made with an unpaired *t* test with Welch's correction. If the data did not comply with normal distribution, Mann–Whitney test was used. For the comparisons of binary variables, Fisher's exact test was used. A *p*‐value of < 0.05 was considered statistically significant.

## 3 Results

### 3.1 Baseline Clinical Characteristics

A total of 129 patients who underwent incision‐thread‐drawing surgery for anal fistula were included in this study. Based on incision healing status at 1‐month post‐surgery, patients were divided into the healing group (*n* = 87), with Grade A incision healing (well‐healed incision without adverse reactions), and the non‐healing group (*n* = 42), with Grade B incision healing (incisional healing with signs of inflammation such as redness, swelling, or fluid accumulation) (Table 1). There were no significant differences between the two groups in terms of age, duration of anal fistula, gender, anal fistula location, or type (*p* > 0.05). However, a significantly higher proportion of patients in the non‐healing group had comorbid diabetes mellitus (40.5% vs. 13.8%, *p* = 0.001), constipation (59.5% vs. 34.5%, *p* = 0.008), and malnutrition (28.6% vs. 12.6%, *p* = 0.047). Hyperlipemia also showed a trend toward significance, being more prevalent in the non‐healing group (38.1% vs. 21.8%, *p* = 0.059). Other factors, such as hypertension, smoking status, and operation time, did not differ significantly between the groups (*p* > 0.05).

**TABLE 1:** Baseline clinical characteristics of different incision healing (Healing and Non‐healing at 1‐month post‐surgery) in patients with anal fistula treated by incision‐thread‐drawing surgery.

Characteristics	Healing (*n* = 87)	Non‐healing (*n* = 42)	*p*
Age (years)	47.93 ± 8.84	49.65 ± 9.72	0.217
Courses of anal fistula (months)	5.63 ± 2.52	6.41 ± 3.03	0.105
Gender			
Male	61 (70.1%)	27 (64.3%)	0.548
Female	26 (29.9%)	15 (35.7%)	
Anal fistula location			
High	57 (65.5%)	26 (61.9%)	0.699
Low	30 (34.5%)	16 (38.1%)	
Anal fistula type			
Simple	58 (66.7%)	23 (54.8%)	0.244
Complex	29 (33.3%)	19 (45.2%)	
Diabetes mellitus			
Yes	12 (13.8%)	17 (40.5%)	0.001
No	75 (86.2%)	25 (59.5%)	
Hypertension			
Yes	24 (27.6%)	14 (33.3%)	0.540
No	63 (72.4%)	28 (66.7%)	
Hyperlipemia			
Yes	19 (21.8%)	16 (38.1%)	0.059
No	68 (78.2%)	26 (61.9%)	
Constipation			
Yes	30 (34.5%)	25 (59.5%)	0.008
No	57 (65.5%)	17 (40.5%)	
Malnutrition			
Yes	11 (12.6%)	12 (28.6%)	0.047
No	76 (87.4%)	30 (71.4%)	
Smoke			
Yes	28 (32.2%)	20 (47.6%)	0.119
No	59 (67.8%)	22 (52.4%)	
Operation time (min)			
< 30	50 (57.5%)	17 (40.5%)	0.091
≥ 30	37 (42.5%)	25 (59.5%)	

*Note:* The data are presented as mean ± SD or *n* (percentage). The comparisons of data were done by Mann–Whitney test or Fisher's exact test.

### 3.2 Preoperative Serum IGF‐1 Levels and Incision Healing

**FIGURE 1:**
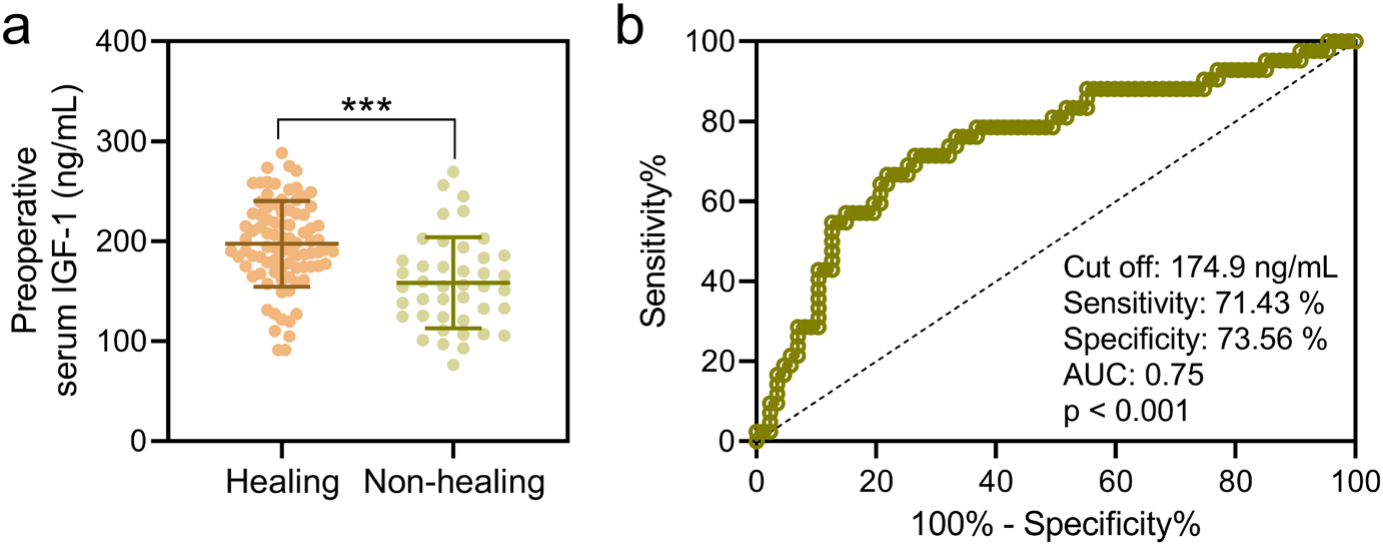
Comparisons of preoperative serum IGF‐1 between different incision healing (healing, *n* = 87, and non‐healing, *n* = 42, at 1‐month post‐surgery) in patients with anal fistula treated by incision‐thread‐drawing surgery. The data are presented as mean ± SD. ***p < 0.001 from unpaired *t* test with Welch's correction. (b) ROC analysis of the predictive value of preoperative serum IGF‐1 for poor incision healing at 1‐month post‐surgery in patients with anal fistula treated by incision‐thread‐drawing surgery.

Preoperative serum IGF‐1 levels were significantly lower in the non‐healing group compared to the healing group (Figure 1a; *p* < 0.001). Receiver operating characteristic (ROC) analysis demonstrated that preoperative serum IGF‐1 has a predictive value for poor incision healing at 1 month post‐surgery. The area under the curve (AUC), cut‐off value, sensitivity, and specificity are shown in Figure 1b. The optimal cut‐off value of preoperative serum IGF‐1 for predicting poor incision healing was 174.9 ng/mL.

### 3.3 Multivariate Analysis of Risk Factors for Poor Incision Healing

Multivariate logistic regression analysis identified diabetes mellitus (OR = 1.993, 95% CI: 1.152–3.524, *p* = 0.007), constipation (OR = 1.648, 95% CI: 1.094–2.895, *p* = 0.015), and operation time greater than 38 min (OR = 1.491, 95% CI: 1.036–2.312, *p* = 0.027) as independent risk factors for poor incision healing at 1‐month post‐surgery (Table 2). Conversely, preoperative serum IGF‐1 levels greater than 174.9 ng/mL were found to be a protective factor for good incision healing (OR = 0.603, 95% CI: 0.347–0.894, *p* = 0.005). Additionally, the serum IGF‐1 levels between 29 diabetic and 100 non‐diabetic patients among the 129 anal fistula patients were compared. It was found that serum IGF‐1 levels were significantly lower in diabetic patients when compared to those in non‐diabetic patients (Figure S1).

**TABLE 2:** Multivariate logistic analysis of clinicopathological factors for poor incision healing at 1‐month post‐surgery in patients with anal fistula treated by incision‐thread‐drawing surgery.

	OR	95% CI	*p*
Diabetes mellitus	1.993	1.152–3.524	0.007
Constipation	1.648	1.094–2.895	0.015
Malnutrition	1.367	0.973–2.448	0.094
Operation time > 38 min	1.491	1.036–2.312	0.027
Preoperative serum IGF‐1 > 174.9 ng/mL	0.603	0.347–0.894	0.005

Abbreviations: CI, confidence interval; OR, odds ratio.

### 3.4 Association of Preoperative Serum IGF‐1 Levels With Postoperative Anal Function

Patients were stratified into low IGF‐1 (*n* = 53) and high IGF‐1 (*n* = 76) groups based on the cut‐off value (174.9 ng/mL) derived from the ROC analysis. In the healing group with 87 cases, 23 cases were stratified into low IGF‐1 and 64 cases were stratified into high IGF‐1. In the non‐healing group with 42 cases, 30 cases were stratified into low IGF‐1 and 12 cases were stratified into high IGF‐1.

**FIGURE 2:**
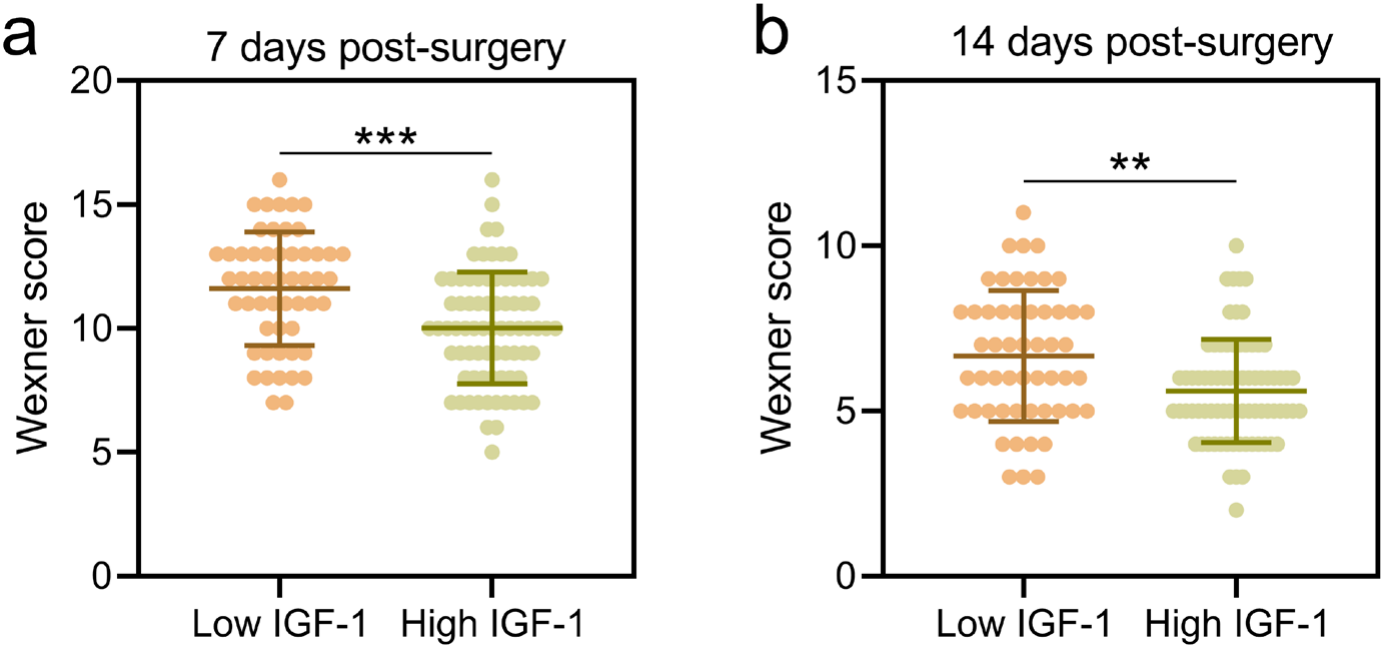
According to the cut‐off in ROC analysis of the predictive value of preoperative serum IGF‐1 for poor incision healing at 1‐month post‐surgery in patients with anal fistula treated by incision‐thread‐drawing surgery, the patients were divided into low IGF‐1 group (*n* = 53) and High IGF‐1 (*n* = 76) group. Comparisons of Wexner score between the two groups at the time of 7 days post‐surgery (a) and 14 days post‐surgery (b). The data are presented as mean ± SD. **p < 0.01 and ***p < 0.001 from unpaired *t* test with Welch's correction.

The Wexner score for anal incontinence, which evaluates postoperative anal function, was significantly lower in the high IGF‐1 group than in the low IGF‐1 group at both 7 days (Figure 2a; *p* < 0.001) and 14 days post‐surgery (Figure 2b; *p* < 0.01). This suggests that higher preoperative serum IGF‐1 levels are associated with better postoperative anal function recovery.

### 3.5 Levels of VEGF, TGF‐β, and Inflammatory Cytokines in Granulation Tissue

**FIGURE 3:**
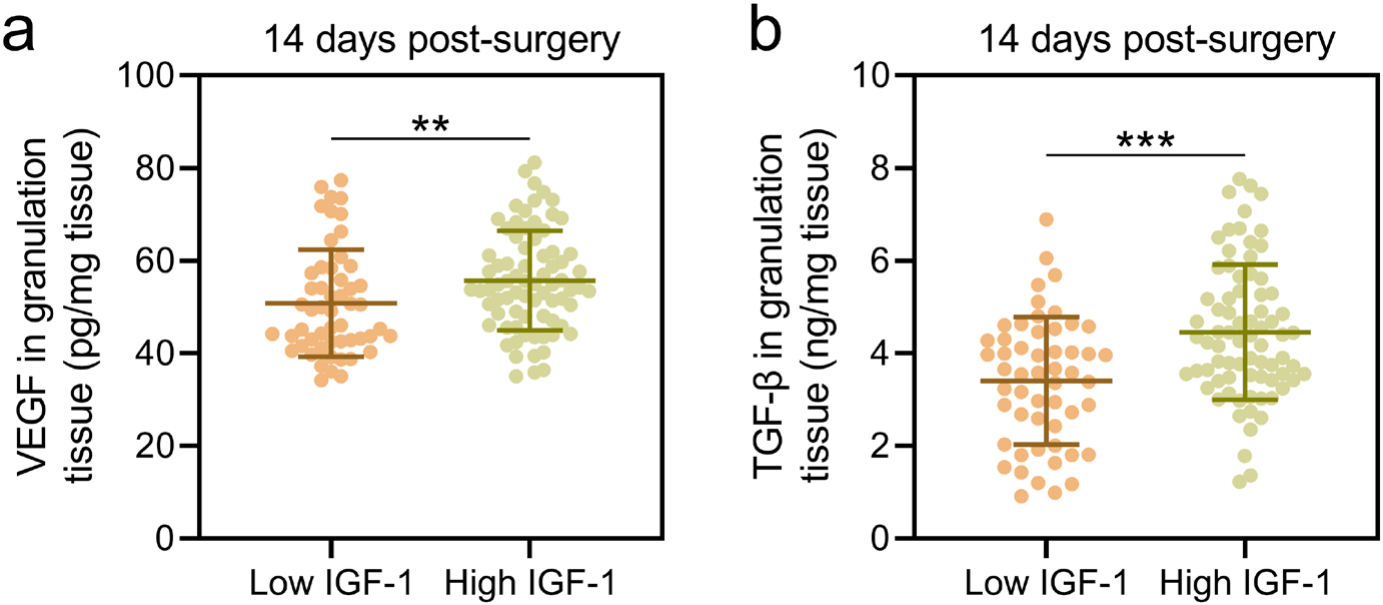
According to the cut‐off in ROC analysis of the predictive value of preoperative serum IGF‐1 for poor incision healing at 1‐month post‐surgery in patients with anal fistula treated by incision‐thread‐drawing surgery, the patients were divided into low IGF‐1 group (*n* = 53) and High IGF‐1 (*n* = 76) group. Comparisons of VEGF level (a) and TGF‐β level (b) in granulation tissues between the two groups at the time of 14 days post‐surgery. The data are presented as mean ± SD. **p < 0.01 and ***p < 0.001 from unpaired *t* test with Welch's correction.

**FIGURE 4:**
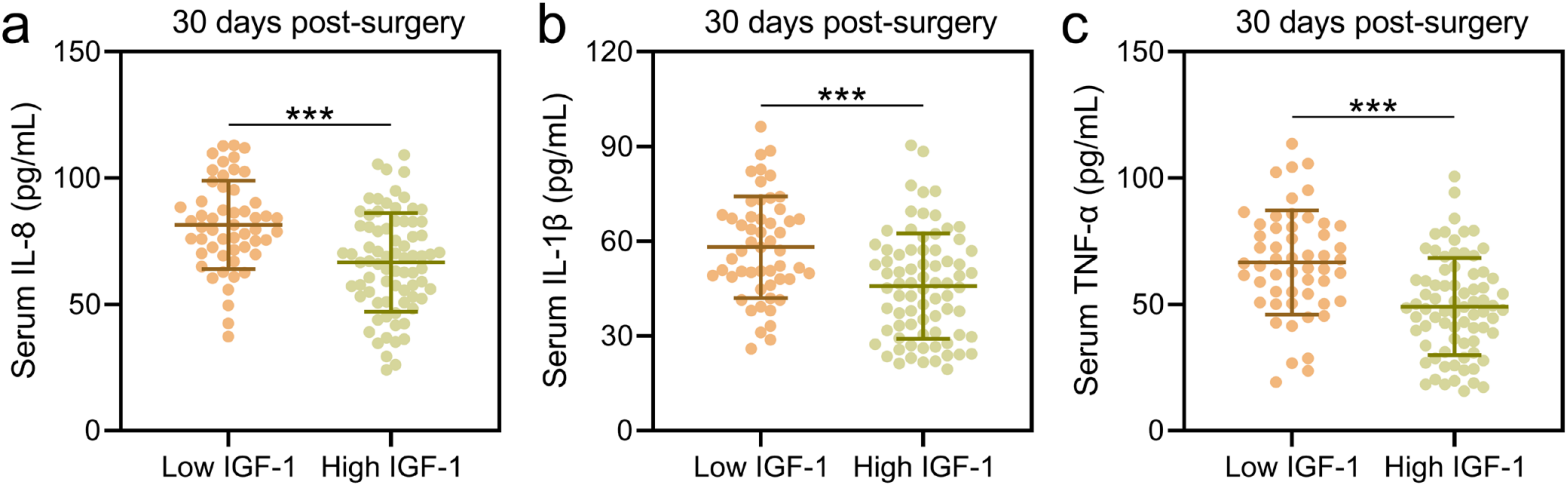
According to the cut‐off in ROC analysis of the predictive value of preoperative serum IGF‐1 for poor incision healing at 1‐month post‐surgery in patients with anal fistula treated by incision‐thread‐drawing surgery, the patients were divided into low IGF‐1 group (*n* = 53) and High IGF‐1 (*n* = 76) group. Comparisons of serum IL‐8 (a), IL‐1β (b) and TNF‐α (c) between the two groups at the time of 30 days post‐surgery. The data are presented as mean ± SD. ***p < 0.001 from unpaired *t* test with Welch's correction.

At 14 days post‐surgery, levels of VEGF and transforming growth factor‐beta (TGF‐β) in granulation tissue were significantly higher in the high IGF‐1 group compared to the low IGF‐1 group (VEGF: Figure 3a; *p* < 0.01; TGF‐β: Figure 3b; *p* < 0.001). Additionally, at 30 days post‐surgery, the concentrations of inflammatory cytokines, including IL‐8, IL‐1β, and TNF‐α, were significantly lower in the high IGF‐1 group compared to the low IGF‐1 group (IL‐8: Figure 4a; IL‐1β: Figure 4b; TNF‐α: Figure 4c; *p* < 0.001). Moreover, it was observed that the preoperative serum levels of IL‐8, IL‐1β, and TNF‐α were significantly higher in the poor‐healing group compared to the healing group (Figure S2a–c). However, when ROC analysis was used to evaluate their predictive value for incision healing, the AUC for all three indicators was relatively low (Figure S2d–f). Additionally, the serum levels of IL‐8, IL‐1β, and TNF‐α in the low IGF‐1 group were higher than those in the high IGF‐1 group (Figure S3a–c), indicating a negative correlation between preoperative serum inflammation levels and IGF‐1 levels. These findings indicate that higher preoperative serum IGF‐1 levels are associated with a more favorable anti‐inflammatory microenvironment, potentially facilitating better wound healing.

## 4 Discussion

This study highlights the prognostic value of preoperative serum IGF‐1 levels in patients undergoing incision‐thread‐drawing surgery for anal fistula treatment. Our findings demonstrate that lower preoperative IGF‐1 levels are associated with poor incision healing and impaired postoperative anal function recovery. Conversely, higher IGF‐1 levels correlate with better surgical outcomes, suggesting that IGF‐1 may serve as a valuable biomarker for predicting healing outcomes in patients with anal fistula undergoing incision‐thread‐drawing surgery. This discussion focuses on the clinical significance of these findings, the potential mechanisms through which IGF‐1 influences wound healing and inflammation, and the implications for clinical practice.

Anal fistulas are challenging to treat due to their complex etiology and the risk of postoperative complications such as infection, recurrence, and fecal incontinence [20]. Traditional surgical approaches, while effective, often come with a high risk of sphincter damage and subsequent incontinence [21]. Incision‐thread‐drawing surgery remains the main treatment for anal fistulas. However, postoperative wound infection and delayed healing remain significant concerns in anal fistula surgery.

Our study demonstrated that preoperative serum IGF‐1 levels are a reliable predictor of wound healing outcomes following incision‐thread‐drawing surgery for anal fistula. The ROC analysis identified a cut‐off value of 174.9 ng/mL for preoperative serum IGF‐1, above which the likelihood of good incision healing increased significantly. Patients with serum IGF‐1 levels above this threshold experienced fewer complications, including lower rates of inflammation (redness, swelling, fluid accumulation), and demonstrated improved postoperative anal function as indicated by lower Wexner incontinence scores. These findings suggest that assessing serum IGF‐1 levels preoperatively could help stratify patients at higher risk for poor healing and inform individualized treatment and postoperative care strategies.

The significantly lower serum IGF‐1 levels in diabetic patients suggest a potential link between insulin resistance and impaired IGF‐1 production. Diabetic patients had significantly lower IGF‐1 levels, likely due to insulin resistance impairing IGF‐1 production. Since IGF‐1 promotes fibroblast proliferation and collagen synthesis, its deficiency may contribute to delayed wound healing [22]. Monitoring and restoring IGF‐1 could be a potential therapeutic strategy for improving healing outcomes in diabetic patients.

The role of IGF‐1 in tissue repair and wound healing is well‐documented, primarily due to its ability to promote cell proliferation, differentiation, and survival [23]. IGF‐1 exerts its effects by binding to the IGF‐1R on cell surfaces, activating downstream signaling pathways such as the PI3K/Akt and MAPK/ERK pathways, which are crucial for cellular growth and survival [11]. In the context of wound healing, IGF‐1 enhances fibroblast proliferation, collagen synthesis, and angiogenesis—key processes required for tissue regeneration and repair.

Our study showed that higher preoperative IGF‐1 levels were associated with elevated levels of VEGF and TGF‐β in granulation tissue at 14 days post‐surgery. VEGF is a potent angiogenic factor that promotes the formation of new blood vessels, essential for delivering oxygen and nutrients to the healing tissue. TGF‐β plays a critical role in modulating inflammation and promoting the deposition of extracellular matrix components, thereby facilitating wound closure. The upregulation of VEGF and TGF‐β in patients with high preoperative IGF‐1 levels may partly explain the improved wound healing observed in this group, suggesting that IGF‐1 supports an environment conducive to effective tissue repair.

Inflammation is a double‐edged sword in wound healing—it is essential for clearing debris and pathogens but if uncontrolled, can lead to excessive tissue damage and fibrosis, impairing wound repair. In our study, patients with higher preoperative IGF‐1 levels exhibited significantly lower levels of pro‐inflammatory cytokines, such as IL‐8, IL‐1β, and TNF‐α, in wound granulation tissue at 30 days post‐surgery. This finding indicates that IGF‐1 may also exert anti‐inflammatory effects that help modulate the wound healing process.

IGF‐1's anti‐inflammatory properties may be attributed to its ability to influence macrophage polarization. Macrophages are key immune cells that play a pivotal role in the inflammation and resolution phases of wound healing. IGF‐1 has been shown to promote the transition from a pro‐inflammatory M1 macrophage phenotype to a pro‐healing M2 phenotype, which secretes anti‐inflammatory cytokines and growth factors that support tissue repair. This shift in macrophage polarization may help reduce excessive inflammation, prevent chronic wounds, and promote a balanced healing response.

Higher IL‐8, IL‐1β, and TNF‐α levels in the poor‐healing group suggest that excessive inflammation hinders wound healing. However, low AUC values from ROC analysis indicate these markers alone are weak predictors of incision healing. A comprehensive approach integrating multiple biomarkers may improve risk assessment. IGF‐1 negatively correlates with pro‐inflammatory cytokines, suggesting its role in creating a favorable healing environment. Higher IGF‐1 levels may suppress excessive inflammation and enhance tissue repair [24]. This supports IGF‐1 as a potential biomarker and therapeutic target for improved postoperative recovery.

The findings from this study have several important implications for clinical practice in the management of anal fistula. First, preoperative measurement of serum IGF‐1 levels could become a standard part of the preoperative assessment for patients undergoing anal fistula surgery. Identifying patients with low IGF‐1 levels could allow for targeted interventions, such as optimizing nutrition, managing comorbidities (e.g., diabetes mellitus), or using pharmacological agents to modulate IGF‐1 levels before surgery. Second, for patients identified as being at high risk for poor healing (e.g., those with low IGF‐1 levels), more intensive postoperative monitoring and care may be warranted. Strategies could include closer follow‐up visits, use of prophylactic antibiotics, or application of adjunctive therapies such as growth factor dressings or topical agents to enhance local IGF‐1 activity and support healing. Third, these findings open up new avenues for research into therapeutic modulation of IGF‐1 levels in patients with anal fistula. While IGF‐1 replacement therapy is not currently standard practice, future studies could explore the feasibility and efficacy of such an approach in patients with persistently low serum IGF‐1 levels. Moreover, understanding the molecular mechanisms by which IGF‐1 influences wound healing and inflammation could lead to the development of novel therapeutics that mimic or enhance its effects.

Several limitations should be acknowledged. While we explored the association between IGF‐1 levels and wound healing outcomes, we did not investigate the potential impact of various factors, such as nutritional status, medication use, or genetic predisposition, on IGF‐1 levels. Future research should aim to elucidate these factors and to explore whether interventions targeting these can modify IGF‐1 levels and improve clinical outcomes.

## 5 Conclusions

In conclusion, this study underscores the clinical significance of preoperative serum IGF‐1 levels as a prognostic marker for wound healing in patients undergoing incision‐thread‐drawing surgery for anal fistula. By integrating IGF‐1 measurement into preoperative planning, clinicians can better stratify patients based on their risk for poor healing and tailor their surgical and postoperative management accordingly. Further research is warranted to explore therapeutic strategies that target IGF‐1 pathways, potentially offering new solutions to enhance recovery and minimize complications in anal fistula surgery.

## Author Contributions

**Zhijun Wu:** data curation, validation, writing – original draft, writing – review and editing. **Xuexue Yin:** data curation, validation, writing – original draft, writing – review and editing. **Jing Li:** data curation, funding acquisition, resources, supervision, validation, writing – original draft, writing – review and editing.

## Ethics Statement

The study was approved by Zibo Central Hospital. Written informed consent was obtained from the patients.

## Consent

The authors have nothing to report.

## Conflicts of Interest

The authors declare no conflicts of interest.

## Supporting information


**Figure S1.** Comparisons of preoperative serum IGF‐1.
**Figure S2.** Comparisons of preoperative serum IL‐8, IL‐1β and TNF‐α between different incision healing in patients.
**Figure S3.** Comparisons of preoperative serum IL‐8, IL‐1β and TNF‐α in low and high IGF‐1 groups.

## Data Availability

The raw data could be obtained upon reasonable request to the corresponding author.
