# Supplementary material for: Prognostic Value of Serum Insulin‐Like Growth Factor‐1 in Patients With Anal Fistula Treated by Incision‐Thread‐Drawing Surgery
Source: Ann Gastroenterol Surg. 2025 May 7;9(6):1362–8. doi: 10.1002/ags3.70037 (PMC12586943; doi:10.1002/ags3.70037)
Supplement: Supplementary file 1 — Figure S1. Comparisons of preoperative serum IGF‐1. Figure S2. Comparisons of preoperative serum IL‐8, IL‐1β and TNF‐α between different incision healing in patients. Figure S3. Comparisons of preoperative serum IL‐8, IL‐1β and TNF‐α in low and high IGF‐1 groups. [file AGS3-9-1362-s001.docx]

Supporting Information


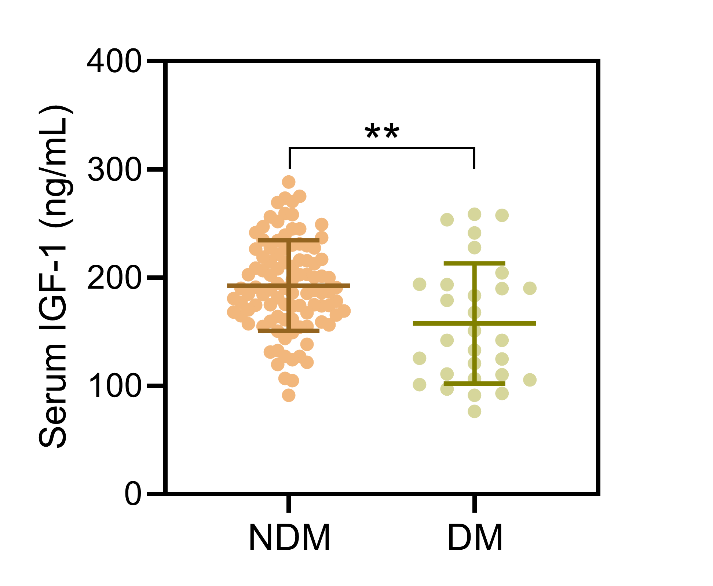


Figure S1. Comparisons of preoperative serum IGF-1 between patients without Diabetes mellitus (NDM, n = 100) and with Diabetes mellitus (n = 29) in patients with anal fistula treated by incision-thread-drawing surgery. The data are presented as mean ± SD. ** p < 0.01 from Unpaired t test with Welch's correction.


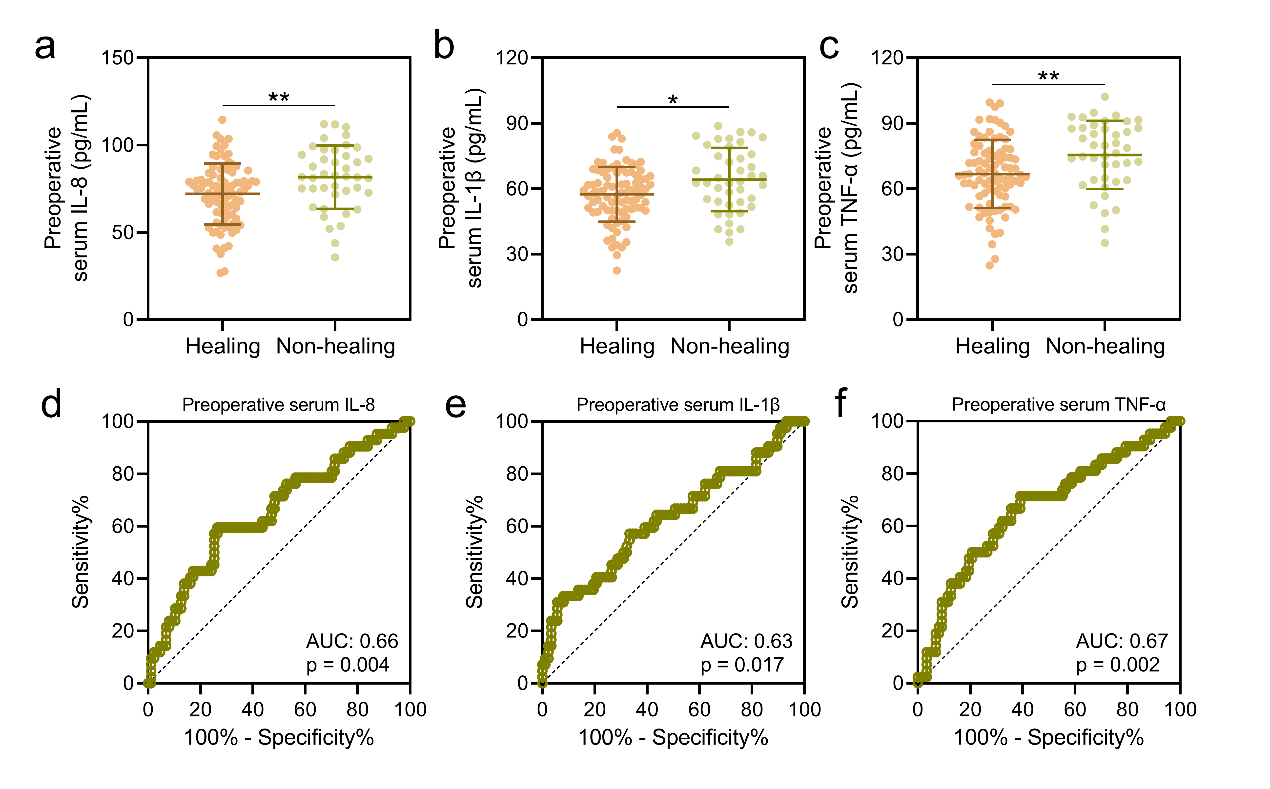


Figure S2. Comparisons of preoperative serum IL-8 (a), IL-1β (b) and TNF-α (c) between different incision healing (Healing, n = 87, and Non-healing, n = 42, at 1-month post-surgery) in patients with anal fistula treated by incision-thread-drawing surgery. The data are presented as mean ± SD. * p < 0.05, ** p < 0.01 from Unpaired t test with Welch's correction. ROC analysis of the predictive value of preoperative serum L-8 (d), IL-1β (e) and TNF-α (f) for poor incision healing at 1-month post-surgery in patients with anal fistula treated by incision-thread-drawing surgery.


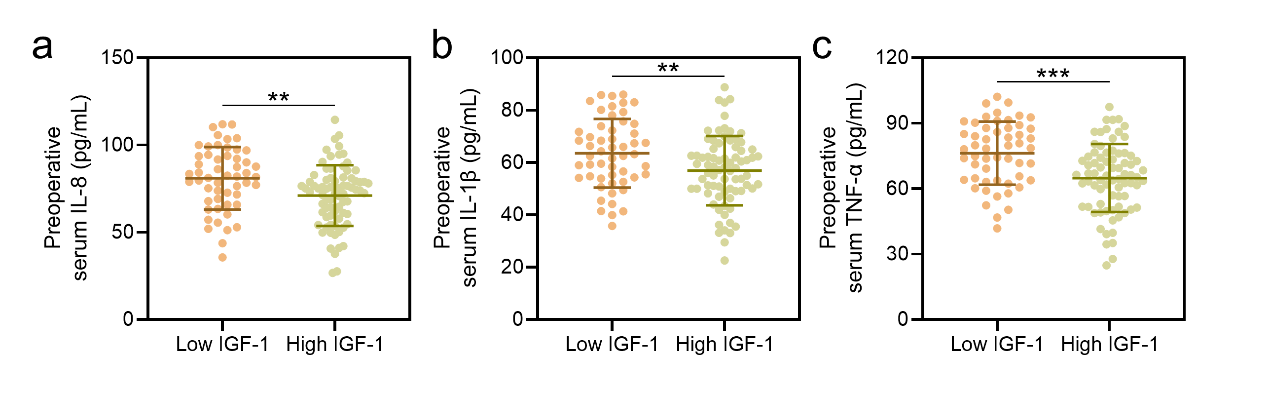


Figure S3. Comparisons of preoperative serum IL-8 (a), IL-1β (b) and TNF-α (c). According to the cut off in ROC analysis of the predictive value of preoperative serum IGF-1 for poor incision healing at 1-month post-surgery in patients with anal fistula treated by incision-thread-drawing surgery, the patients were divided into Low IGF-1 group (n = 53) and High IGF-1 (n = 76) group. The data are presented as mean ± SD. ** p < 0.01, *** p < 0.001 from Unpaired t test with Welch's correction.
